# Innovative Colorimetric Neutral Red-Based Loop-Mediated Isothermal Amplification (NR-LAMP) Assay: Transforming Rapid and Affordable Feline Leukemia Virus Detection

**DOI:** 10.3390/ijms262411793

**Published:** 2025-12-05

**Authors:** Witsanu Rapichai, Piyamat Khamsingnok, Anyalak Wachirachaikarn, Thawee Laodim, Hieu Van Dong, Nianrawan Meecharoen, Siriluk Ratanabunyong, Thanawat Khaoiam, Supansa Tuanthap, Amonpun Rattanasrisomporn, Selapoom Pairor, Kiattawee Choowongkomon, Natthasit Tansakul, Peter A. Lieberzeit, Jatuporn Rattanasrisomporn

**Affiliations:** 1 Center for Advanced Studies for Agriculture and Food, Kasetsart University Institute for Advanced Studies, Kasetsart University, Bangkok 10900, Thailand; tswitsanu@gmail.com; 2 Department of Companion Animal Clinical Sciences, Faculty of Veterinary Medicine, Kasetsart University, Bangkok 10900, Thailand; piyamat.kha@ku.th (P.K.); thanawat.khao@ku.th (T.K.); selapoom_ake@hotmail.com (S.P.); 3 Department of Biochemistry, Faculty of Science, Kasetsart University, Bangkok 10900, Thailand; ae.med@hotmail.com (S.R.); fsciktc@ku.ac.th (K.C.); 4 Department of Sciences and Bioinnovation, Faculty of Liberal Arts and Science, Kasetsart University, Kamphaeng Saen Campus, Nakhon Pathom 73140, Thailand; faasalw@ku.ac.th; 5 Department of Animal Science, Faculty of Agriculture at Kamphaeng Saen, Kasetsart University, Kamphaeng Saen Campus, Nakhon Pathom 73140, Thailand; fagrtwl@ku.ac.th; 6 Faculty of Veterinary Medicine, Vietnam National University of Agriculture, Trau Quy Town, Gia Lam District, Hanoi 131000, Vietnam; dvhieuvet@vnua.edu.vn; 7 Central Laboratory (CTL), Center for Veterinary Research and Innovation, Faculty of Veterinary Medicine, Kasetsart University, Bangkok 10900, Thailand; nianrawan.mee@ku.ac.th; 8 Kasetsart University Veterinary Teaching Hospital, Faculty of Veterinary Medicine, Kasetsart University, Kamphaeng Saen Campus, Nakhon Pathom 73140, Thailand; 9 Faculty of Veterinary Medicine, Rajamangala University of Technology Tawan-ok, Bangpra, Chonburi 20110, Thailand; supansa_tu@rmutto.ac.th; 10 Interdisciplinary of Genetic Engineering and Bioinformatics, Graduate School, Kasetsart University, Bangkok 10900, Thailand; fgraapr@ku.ac.th; 11 Department of Pharmacology, Faculty of Veterinary Medicine, Kasetsart University, Bangkok 10900, Thailand; natthasit.t@ku.th; 12 Department of Physical Chemistry, Faculty of Chemistry, University of Vienna, Waehringer Strasse 42, A-1090 Vienna, Austria; peter.lieberzeit@univie.ac.at

**Keywords:** feline leukemia virus (FeLV), colorimetric LAMP, neutral red, *pol* gene, cost-effective clinical application

## Abstract

Feline leukemia virus (FeLV) is a retrovirus that globally affects both domestic and wild cats, leading to the development of leukemia, lymphoma, and immunosuppression. However, it is important to note that the daily antigen test may yield false negative results. In this study, we successfully developed the first colorimetric loop-mediated isothermal amplification (LAMP) associated with neutral red (NR-LAMP) for the detection of FeLV. The NR-LAMP assay exhibited high sensitivity and efficiency compared to the routine polymerase chain reaction (PCR) reference method. To ensure specificity, a novel LAMP primer set was custom-designed based on the *pol* gene of multiple FeLV strains, which resulted in no cross-amplification with other feline viruses. Under the optimized isothermal amplification conditions at 61 °C for 40 min, the NR-LAMP assay achieved a detection limit of 10^0^ copies/µL. Using a blind clinical test involving 98 samples, the NR-LAMP assay demonstrated perfect agreement with the reference PCR method, providing a sensitivity of 97.3% and a specificity of 100%. This proposed NR-LAMP assay surpasses other related approaches in terms of sensitivity, efficiency, and cost-effectiveness. Consequently, the colorimetric NR-LAMP reaction serves as a robust and convenient diagnostic tool for the inspection of FeLV, offering an alternative molecular method for future clinical applications and commercial utilization.

## 1. Introduction

Feline leukemia virus (FeLV) belongs to the *Gammaretrovirus* genus within the *Retroviridae* family [1]. The viral genome consists of a linear single-stranded RNA of approximately 8.3 kb in length, containing two open reading frames (ORFs) with long terminal repeats (LTRs) flanking them. In gammaretroviruses such as FeLV, *gag* and *pol* lie in the same reading frame; the in-frame UAG stop codon between them as glutamine permits a minority of ribosomes to translate into the *pol* ORF [1,2]. For its structural protein-encoding genes, *gag* gene encodes for p15 matrix (MA), p12, p27 capsid (CA), and p10 nucleocapsid (NC), whereas *env* gene encodes for gp70 surface glycoprotein (SU) and p15E transmembrane protein (TM) [1]. The *pol* gene encodes for the nonstructural proteins p14 protease (PR), p80 reverse transcriptase (RT), and p46 integrase (IN) [1].

Feline leukemia virus (FeLV) acts as an infectious agent that is capable of causing hematological disorders, immunodeficiency, macrocytic anemia, aplastic anemia, leukemia, and lymphoma in domestic cats [3]. Viral infection frequently results in the fatality of domestic cats worldwide [4]. FeLV was globally reported in European [5,6,7], American [8,9], Asian countries [10,11,12], Australia [13], and New Zealand [14]. Regarding infection in cats, this virus is typically transmitted horizontally through saliva or nasal secretion contact among cats living together or through cat fighting and also transmitted vertically from maternal cats to their kittens [15,16]. Modes of FeLV infection have been categorized into three phases based on provirus, plasma RNA, and antibody-reactive progressive infection. No FeLV provirus or viral RNA can be found in two situations: cats that have anti-FeLV antibodies after a small virus dose and have cleared the infection, and cats that have never been exposed and have no antibodies [16,17,18]. Regressive infection is marked by short-lived viremia and only a fleeting appearance of FeLV antigen in the blood. During this phase, the viral RNA is copied into DNA, which slips into the cat’s own genome to form a provirus. If this provirus later inserts into reproductive cells, it can be passed from mother to kitten as true endogenous FeLV. Stress or any event that weakens immunity can re-activate the silent provirus, so regressor cats may suffer recurrent bouts of disease [16,18]. Once the virus has replicated widely and spread to lymphoid tissues, bone marrow, and the lining of mucous membranes and glands, cats enter the progressive stage of FeLV infection. At this point, high levels of virus persist in the blood, and these cats become the main source of infection for others [16,18,19].

To control the disease, vaccination against FeLV has been developed for domestic cats [20,21,22]. In addition, the isolation of infected cats from healthy animals is necessary to reduce the risk of infection. Since clinical signs of diseased cats infected with FeLV are not specific, laboratory methods are necessary to detect FeLV infection in disease cases. Rapid immunochromatography is typically used to detect the p27 antigen for routine FeLV infection diagnosis. Even though this assay can detect the stage of viremia or the presence of antibodies in the blood and other kinds of secretion, early viremia and latent infections might produce false negative results. This also happens when viruses are detected using ELISA, immunofluorescence, and virus isolation techniques [16]. PCR-based methods including conventional PCR and real-time PCR have been developed to identify FeLV proviral DNA in tissue, plasma, or secretions [23,24]. Nested PCR for FeLV detection based on a highly conserved region of the *pol* gene sequence was developed [25]. The method was reported to be highly sensitive, specific, fast, and convenient [25]. Real-time PCR also indicated high sensitivity and specificity and was highly recommended for assessing the progress of viral infection and quantification of viral load. However, these PCR methods are time-consuming and costly since sophisticated laboratory facilities are needed. Loop-mediated isothermal amplification (LAMP), which has been reported as a rapid, simple, and highly specific method [26,27,28], can be exploited as another choice for FeLV detection.

The loop-mediated isothermal amplification (LAMP) technique is a rapid nucleic acid amplification method that operates at a constant temperature, typically between 60 and 65 °C, thereby eliminating the dependency on the expensive thermal cyclers required for PCR. Originally developed in 2000 by Notomi and colleagues, the method utilizes a strand-displacing activity of DNA polymerase (such as *Bst* DNA polymerase) and a set of four to six specially designed primers that recognize six to eight distinct regions on the target DNA sequence [27]. This sophisticated primer design initiates the synthesis of stem-loop DNA structures, which subsequently serve as templates for auto-cycling amplification, leading to the generation of long DNA concatemers. A key byproduct of this massive DNA synthesis is a stoichiometric, mass release of pyrophosphate ions. This allows for detection through various means, including a visible increase in turbidity [29], a fluorescence shift using indicators like calcein-Mn^2+^ [30], or real-time monitoring with intercalating dyes [31,32]. Capable of detecting as few as ten copies of the target template within just 30–60 min, LAMP is significantly faster, more robust for field use, and less instrument-reliant than conventional PCR. These attributes firmly establish it as a frontline tool for point-of-care diagnosis of human, animal, and plant pathogens. Continuous advancements, particularly in multiplexing capabilities and the development of simple colorimetric or visual readouts, are further extending its application into resource-limited settings globally [28,31,32,33].

In this study, a simple, convenient, cost-effective, sensitive, and specific LAMP assay based on the *pol* gene sequence of FeLV was developed. This work represents an alternative means of efficient detection and of monitoring FeLV infection for clinical use.

## 2. Results

### 2.1. Optimization of Amplification Temperatures

**Figure 1:**
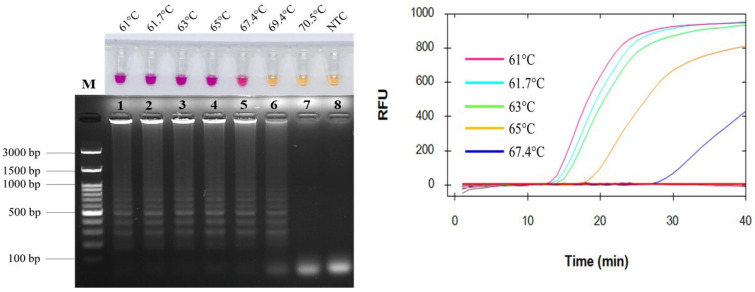
Optimization of temperatures by real-time LAMP assay and by agarose gel electrophoresis. Pink color indicates a positive reaction, and yellow color indicates a negative one. NTC, negative control. Lane M, 100 bp Ladder DNA Marker III (Yeastern Biotech, New Taipei City, Taiwan); lane 1–7, DNA band pattern amplified at 61, 61.7, 63, 65, 67.4, 69.4, and 70.5 °C; lane 8, NTC.

A neutral red-based FeLV LAMP assay was standardized to verify optimum concentrations of MgCl_2_ and dNTPs. The electrophoretic lane with the DNA band pattern corresponded to the pink-colored appearance of 6 mM MgCl_2_ and 1.4 mM dNTPs, which were then chosen for further experiments (Figures S1 and S2). Different temperatures ranging from 61 to 61.7, 63, 65, 67.4, 69.4, and 70.5 °C were optimized for FeLV LAMP detection via a real-time PCR instrument for 40 min of amplification. Using neutral red (NR) dye as an indicator, the positive reaction was visible to the naked eye, changing the color from yellow to pink to represent the quantity of produced protons released during the LAMP process. Figure 1 clearly shows that 61, 61.7, 63, 65, and 67.4 °C do not only result in pink color, i.e., positive results, but also consistent amplified fluorescent signals. The inconsistent result at 69.4 °C only shows a DNA band pattern, whereas that at 70.5 °C results in a negative reaction. Among the pink color reactions, the fastest amplification rate at 61 °C was chosen as the optimum temperature and applied for all subsequent experiments.

### 2.2. Specificity of Colorimetric LAMP for FeLV Detection

**Figure 2:**
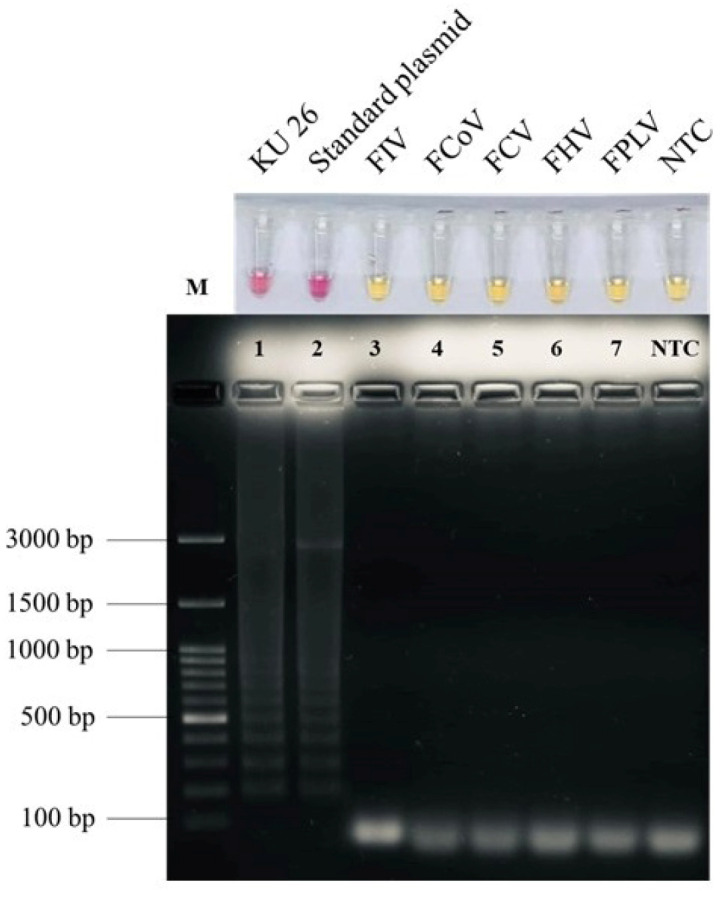
Specificity analysis of FeLV LAMP assay. Upper row shows specific LAMP reaction, and lower row shows AGE analysis. Pink color indicates a positive reaction, and yellow color indicates a negative one. Lane M, 100 bp Ladder DNA Marker III (Yeastern Biotech, New Taipei City, Taiwan); lane 1–2, proviral DNA sample no. KU 26 and standard plasmid harboring *pol* gene FeLV; lane 3–7, multiple feline viral genomes including FIV, FCoV, FCV, FHV, and FPLV; lane NTC, negative control.

The LAMP specificity of the FeLV primer set based on the *pol* gene was investigated using a clinically proviral DNA sample, a recombinant plasmid harboring the *pol* gene, and viral genomic materials of FeLV, FIV, FCoV, FCV, FHV, and FPLV. As shown in Figure 2, our proposed colorimetric LAMP assay does not show cross-reactivity with other feline viruses, except for the proviral DNA of the clinical FeLV sample, KU 26, which showed a pink color that corresponded to that of the standard plasmid. Based on the agarose gel electrophoresis result for LAMP amplicons, the specificity of the FeLV LAMP primer was also confirmed: only DNA KU 26 showed a DNA band pattern as a positive standard plasmid. In addition, the lower DNA ladder band in lane 1 was isolated from the agarose gel and sent for Sanger method sequencing to confirm the identity of the positive LAMP product. The sequencing result revealed that the *pol* gene sequence of the feline leukemia virus (KR030147) shared 98% identity (Figure S3), which was indicative of the specificity of the designed LAMP primer for FeLV detection.

### 2.3. Detection Limit of Colorimetric LAMP for FeLV Detection

**Figure 3:**
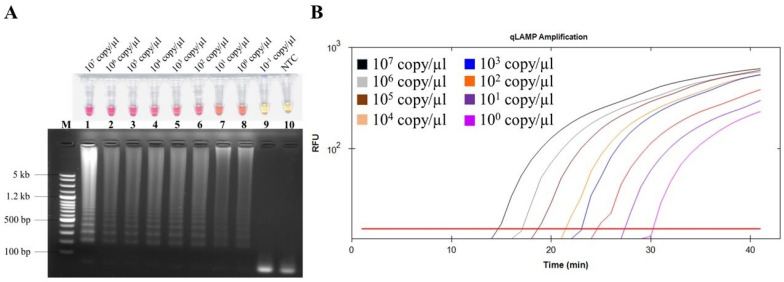
The detection limit of FeLV LAMP assay. (**A**) Upper row shows visual inspection of LAMP reaction with various standard plasmid copy numbers. Pink color indicates positive reaction, and yellow color indicates negative reaction. NTC, negative control. Lower row shows agarose gel electrophoresis results with DNA band pattern. Lane M, 100 bp Ladder DNA Marker IV (Yeastern Biotech, New Taipei City, Taiwan); lane 1–10, LAMP amplicons of 10-fold serially diluted recombinant pGEMT::*pol* gene at 10^7^ copy/µL, 10^6^ copy/µL, 10^5^ copy/µL, 10^4^ copy/µL, 10^3^ copy/µL, 10^2^ copy/µL, 10^1^ copy/µL, 10^0^ copy/µL, 10^−1^ copy/µL, and NTC. (**B**) Relative fluorescent signals plotted with time to positive. Each color line representing different diluted concentrations of recombinant pGEMT::*pol* gene was determined by a real-time cycler instrument.

In order to determine the LAMP detection limit, the standard recombinant plasmid, pGEMT::*pol* gene, was serially diluted 10-fold followed by each sample being templated in an NR-based isothermal reaction. As shown in Figure 3, the detection limit of the proposed colorimetric FeLV LAMP assay is 10^0^ copies/µL. According to the calibration curve, the correlation coefficient (R^2^) value of the quantitative NR-LAMP assay is 0.995, indicative of good linear correlation between the diluted plasmid copy number and fluorescent signal (C_q_ value) (Figure S4). Each positive diluted concentration is also in good agreement, exposing the DNA band pattern with AGE.

### 2.4. LAMP Performance with Clinical Samples and Cost Estimation

To validate the proposed method with clinical samples, blind tests with randomized cat blood samples for inspecting FeLV cats were performed. The diagnostic performance of this technique was statistically estimated and compared with the routine reference method, conventional PCR. As summarized in Table 1, 71 of the 98 clinical samples gave positive LAMP results. Two samples with negative LAMP results yielded positive results when tested with PCR, while 25 gave negative results with both tests. The sensitivity and specificity of LAMP were 97.3% (with 95% confidence intervals (CIs) of 91.8–99.5) and 100% (with 95% CIs of 84.1–99.8), respectively, when compared to conventional PCR. The positive predictive value (PPV) of this approach was 100 (with 95% CIs of 94–99.9), and the negative predictive value (NPV) was 92.6 (with 95% CIs of 78.8–98.7). The kappa coefficient (k) value of 0.9 obtained by the LAMP described here clearly demonstrates that the colorimetric approach has great potential to be utilized together with traditional PCR for FeLV inspection. So far, PCR-based nucleic acid amplification techniques have been adopted as reliable tools for FeLV analysis. Previously, several multiple-temperature amplification methods used to detect the LTR region of leukemia virus in cats have been reported. Certain pros and cons of the developed colorimetric LAMP were compared with other related molecular approaches to detecting FeLV (Table 2). It can be seen that NR-LAMP had the lowest detection limit of 10^0^ copies/µL and can take as little as 40 min to amplify the viral target gene, except for the RPA method, which requires only 10 min for amplification. Moreover, the sensitivity and specificity of RT-qPCR methods and the RPA method were lower than that of NR-LAMP from our work. Hence, NR-LAMP has the potential to be applied in clinics. Afterward, we conducted a cost breakdown for NR-LAMP (Table S1) and compared the expenses associated with the necessary consumables for NR-LAMP and routine PCR (Table 3) by taking into account the prices (in US dollars) of procuring reagents in Thailand during the study period. Our NR-LAMP assay was estimated to cost USD 1.04 per the reaction. In contrast, Kasetsart University Veterinary Teaching Hospital and Private Veterinary Hospital charge USD 17.79 and USD 29.66, respectively, for a PCR test. Thus, NR-LAMP is roughly 20 to 30 times cheaper than the reference method.

## 3. Discussion

Feline leukemia virus (FeLV), a γ-retrovirus belonging to the *Retroviridae* family, was first discovered in a cluster of cats with lymphosarcoma in 1964 [40]. FeLV is capable of transmission through close contact, biting, and shedding through secretion and excretion [41]. It is also vertically transmitted from infected queens to their kittens [16]. Cats with FeLV should be examined and separated from other healthy cats to prevent the spreading of viral transmission. The American Association of Feline Practitioners (AAFP) has recommended screening all cats for FeLV infection at the time of acquisition, before receiving initial vaccination against FeLV, after a possible exposure to infected cats, or if clinical symptoms of the disease appear [16]. Certain diagnostic techniques, such as enzyme-linked immunosorbent assay (ELISA) or rapid immunomigration (RIM), are customarily used to analyze soluble FeLV p27 antigen in whole blood, serum, or plasma [42]. As for, p27 antigen testing is not feasible and shows inaccurate sensitivity for detecting viremia in the progressive stage. Therefore, nucleic acid amplification-based methods with high sensitivity and specificity for detecting FeLV provirus have been recommended as a routine diagnostic tool [16,39]. In particular, loop-mediated isothermal amplification (LAMP), an accurate, rapid, specific, and sensitive technique, has been developed to amplify pathogenically genetic substances at a single temperature with the use of six primers [43]. LAMP products were also straightforward to visualize using a fluorescent marker, a metal-sensitive dye, and a high-contrast pH-sensitive dye [43,44,45,46]. In addition, this LAMP technique has been the most favorable tool for detecting African swine fever virus [47], feline coronavirus [28,48,49,50], Chikungunya virus [51], SARS-CoV-2 [52,53], *Penicillium* species [54], species-specific of *P*. *expansum* [55], *Photobacterium* spp. [56], *Salmonella* in food [57], and food-borne mycotoxigenic fungi [58]. In this regard, we developed a neutral red-based colorimetric LAMP (NR-LAMP), which can detect FeLV proviral DNA from cat whole blood, and we conducted tests to further compare its performance with routine conventional PCR. This showed that the certified NR-LAMP assay could serve as a robust FeLV detection method in the clinical laboratory and for household cat screening for FeLV. In addition, the color changes in the neutral red dye, from a yellow color to a pink color, in the presence of amplified DNA has major advantages: it is easy to observe without the risk of post-contamination by reopening the tube and post-analysis using AGE [28,54,55,59].

The utilization of neutral red (NR) as a pH-sensitive indicator in our LAMP assay represents a significant advancement in colorimetric and fluorometric detection strategies for FeLV. As demonstrated in the optimization and sensitivity evaluations, NR initially presents a yellow color in its deprotonated form, which is compatible with the alkaline environment optimal for *Bst* DNA polymerase activity. This baseline state ensures efficient initiation of the amplification process without compromising enzymatic function. During the LAMP reaction, the generation of protons (H^+^) as a by-product leads to progressive acidification of the reaction mixture, protonating NR and inducing a visible color transition to a pink color. This pH-dependent shift not only enables naked-eye detection but also correlates directly with amplicon yield, enhancing the assay’s practicality for field or resource-limited settings. The modulation of solution pH underscores NR’s dual role as both a visual and quantitative marker, addressing limitations in traditional dyes that may require additional instrumentation. To extend NR-LAMP’s utility for quantitative fluorescence monitoring, the selection of an appropriate reporter dye is critical. Xu et al. (2016) reported that the protonation of NR under acidic conditions enhances fluorescence intensity, accompanied by a red shift in the emission peak from 612 nm to 626 nm [58]. This spectral change aligns closely with the absorbance peak of Texas Red at 615 nm [59], facilitating efficient energy transfer and signal capture. Consequently, Texas Red was preferentially adopted over SYBR Green, which lacks compatibility in the red spectrum and may suffer from non-specific binding issues. This strategic integration improves assay sensitivity and specificity, as evidenced by our detection limit of 10^0^ copies/µL. By leveraging NR’s protonation dynamics, our method offers a cost-effective, robust alternative to conventional PCR, with potential for broader applications in veterinary diagnostics. Future studies could explore multiplex capabilities or integration with portable devices to further enhance point-of-care utility.

In the empirical analysis, the LAMP primer set was designed based on comprehending the *pol* gene sequences of exogenous and endogenous FeLV as previously reported [60]. The LAMP primer set consists of the F3/B3 outer primers, FIP/BIP internal primers, and LF/LB loop primers, which are necessary for our single-step amplification system. In order to maximize LAMP performance, essential parameters to optimize include MgCl_2_ concentrations, dNTP concentrations, and amplification. The Mg^2+^ ion plays a crucial role in increasing template–primer binding affinity and DNA polymerase function in nucleic acid amplification reactions [61]. Several works stated that it was one of the most essential components in optimizing their LAMP applications [62,63,64,65]. For instance, Nie reported 4 mM Mg^2+^ ion, at least, was most suitable for DNA-band pattern appearance [66]. In our NR-LAMP assay, we also established that Mg^2+^ ion was a key factor affecting NR-LAMP performance and the optimal Mg^2+^ ion concentration was 6 mM, showing a clear pink color with a ladder-like DNA band pattern (Figure S1). Consequently, dNTPs are considered to be most indispensable for sugar–phosphate backbone formation since the α-phosphate group is nucleophilic-attacked with a hydroxy group at the 3’-end DNA strand, yielding nucleotide substitution, DNA strand elongation, and proton generation [67]. As seen in Figure S2, a lower dNTPs concentration, 1 mM, yields a DNA band pattern. However, it is inadequate for protonated NR color development. Hence, it has been proven that 1.4 mM dNTPs are the most suitable for our system, resulting in a distinct pink color with DNA ladder-like patterns. LAMP amplification efficiency performed well at a relatively constantly high temperature, unlike PCR, which is carried out at varying temperatures using a sophisticated instrument. An optimal temperature for an isothermal enzyme function is required [65,68]. Incubation at low temperatures might also lead to the potentially non-specific binding of primers to DNA and unspecific results [53]. When compared to conventional PCR, the NR-LAMP test described here is more favorable due to it is more convenient, saves time, and allows for easier detection. In this study, at 61 °C, a colorimetric real-time LAMP reaction yielded optimal results, exhibiting a positively fluorescent signal that appeared within 15 min, which corresponded to a color shift discernable by naked-eye observation and a DNA ladder-like pattern discernable by AGE.

To validate whether the proposed technique could successfully discriminate FeLV from other feline disease-causing viruses, the genomic substances of those viruses isolated from clinical fields were verified using a colorimetric isothermal amplification assay. The positive pink color of the NR-LAMP method developed here did not show cross-reactivity with FIV, FCoV, FCV, FPLV, and FHV, demonstrating high specificity for FeLV. To confirm this, the nucleotide sequence of KU26’s lower DNA band of LAMP product in agarose gel was sequenced. According to the NCBI database, the discovered sequence had 98% similarity to the *pol* gene of feline leukemia virus isolate C85 (accession number KR030147). These findings reveal the identities of the *pol* gene amplification products of the cat leukemia disease-inducing retrovirus. In addition, lacking 100% identity might result from direct sequencing on a relatively small amplification product, as well as the absence of proofreading activity of the isothermal polymerase, which results in a small number of erroneous sequencings in the LAMP product [53].

Among 98 clinical samples, blind testing was conducted on FeLV proviral DNA. The results indicated a strong kappa value of 0.9, demonstrating a perfect correlation between the outcomes obtained using the NR-LAMP method and the routine reference PCR method. Previous studies have reported the sensitivity and specificity of related molecular assays, with RT-qPCR showing 92% sensitivity and 99% specificity [38] and RPA demonstrating 95.89% sensitivity and 100% specificity [39]. Although our described method did not achieve 100% sensitivity, the occurrence of a false-negative outcome could be attributed to the DNA target’s sequence variation and self-designed primers. Given that a larger number of primers were employed during the LAMP reaction, precise nucleotide-to-nucleotide base hybridization becomes crucial in enabling effective amplification of the DNA through strand displacement [69,70]. Our work, however, exhibited a low risk of generating false negative or false positive results. Notably, certain PCR methods that detect the LTR region of the FeLV genome have shown higher limits of detection (LOD) compared to NR-LAMP [35,36,37,38], with the exception of Miyazawa and Jarrett [34], who reported the same LOD value. Our proposed method offers advantages in terms of simplicity, visualization, and a reduced risk of contamination from multi-opening tubes. In contrast, an RPA method with a 10 min amplification time was developed by Lacharoje [39]; however, it had some limitations such as post-amplification detection using AGE, higher reagent prices, fewer available reagent kits, and the use of two enzymes [70,71].

The NR-LAMP assay developed in this study has significant implications for the detection of FeLV proviral DNA. Our method exhibited notable levels of accuracy, specificity, sensitivity, and cost-effectiveness. Furthermore, the colorimetric assay demonstrated promising practical implementation in clinical laboratories. Additionally, the final reaction of the assay enabled straightforward visualization while minimizing the risk of contamination during tube reopening. To the best of our knowledge, this study represents a pioneering utilization of the NR-LAMP approach for FeLV diagnosis. Despite the requirement for proviral DNA extraction, our NR-LAMP assay outperforms other related PCR-based methods in terms of the limit of detection (LOD). Consequently, it possesses several desirable attributes, including simplicity, specificity, rapidity, visual capability, and cost-effectiveness, rendering it a highly promising alternative molecular diagnostic method for FeLV screening in veterinary laboratories and for potential future development in the global market.

## 4. Materials and Methods

### 4.1. Ethical Statements

The National Research Council of Thailand’s Ethical Principles and the Use of Animals for Scientific Purposes criteria were followed when conducting the study. The Animal Care and Use Committee (IACUC) of Kasetsart University, Bangkok, Thailand, gave its approval to the animal use protocol (animal use protocol number ACKU66-VET-046, Date 30 May 2023).

### 4.2. DNA Extraction from Blood Samples

Blood specimens from feline patients were procured at the Veterinary Teaching Hospital of Kasetsart University. Proviral DNA associated with FeLV was isolated from EDTA-anticoagulated whole blood employing the E.Z.N.A. Universal Pathogen Kit (Omega Bio-Tek, Norcross, GA, USA), following the manufacturer’s stipulated protocol. The isolated proviral DNA preparations were subsequently utilized as templates in loop-mediated isothermal Amplification (LAMP) and polymerase chain reaction (PCR) assays.

### 4.3. Recombinant Plasmid Construction

Feline leukemia virus (FeLV) RNA was extracted from the Leukocell^®^2 FeLV vaccine (Zoetis Inc., Parsippany, NJ, USA) employing the E.Z.N.A. Viral RNA Kit (Omega Bio-Tek, Norcross, GA, USA), in strict accordance with the manufacturer’s protocol. This purified RNA served as the template for reverse transcription polymerase chain reaction (RT-PCR) amplification of the *pol* gene, facilitated by the Deoxy+ OneStep RT-PCR kit (Yeastern Biotech, New Taipei City, Taiwan). Primers targeting the *pol* gene were adapted from Ramirez et al. [60] and are depicted in Table 4. The 25 µL RT-PCR mixture consisted of 12.5 µL of 2X OneStep RT-PCR premix, 1.38 µL each of the 10 µM forward (pol-F) and reverse (pol-R) primers, 7.74 µL of RNase-free water, and 2 µL of FeLV RNA template. Reverse transcription was conducted at 42 °C for 60 min, succeeded by enzyme inactivation at 70 °C for 7 min. The ensuing PCR amplification encompassed 45 cycles, each comprising denaturation at 94 °C for 1 min, annealing at 55 °C for 45 s, and extension at 72 °C for 50 s, terminating in a final extension at 72 °C for 10 min [62]. The resultant amplicon, approximately 791 bp, was resolved on a 1.5% (*w*/*v*) agarose gel. The discrete DNA band was meticulously excised and purified utilizing the GeneAll^®^ Expin™ Combo GP kit (GeneAll Research Institute, Seoul, Korea). To construct a standard vector, the purified *pol* gene fragments were ligated into the pGEM-T vector system (Promega, Madison, WI, USA) following the prescribed guidelines, yielding the recombinant plasmid pGEM-T::*pol* gene. This recombinant plasmid was then transformed into competent *Escherichia coli* DH5α cells. Plasmid DNA from recombinant white colonies was isolated using the E.Z.N.A. Plasmid DNA Mini Kit (Omega Bio-Tek, Norcross, GA, USA), adhering to the provided instructions. The refined recombinant plasmid was subsequently utilized as the reference standard for assays assessing optimization, specificity, and sensitivity.

### 4.4. LAMP Primer Design

**Figure 4:**
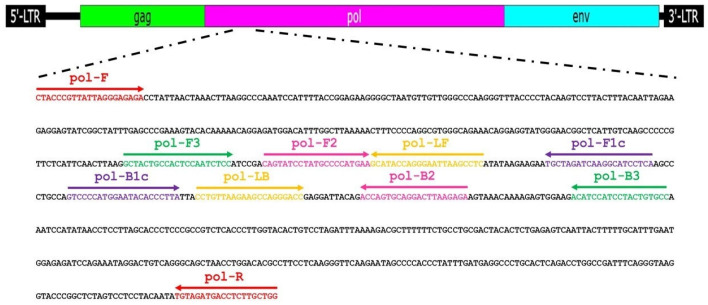
Position of LAMP primers on the 230 bp *pol* gene fragment of FeLV (GenBank accession number NC_001940).

A set of six primers, encompassing the outer primers (pol-F3 and pol-B3), loop primers (pol-LF and pol-LB), and inner primers (pol-FIP and pol-BIP), was meticulously designed based on the FeLV *pol* gene sequence (petty patent number 2503004697). The positions and sequences of these primers are elegantly illustrated in Figure 4 and comprehensively detailed in Table 4.

### 4.5. LAMP Reaction

**Figure 5:**
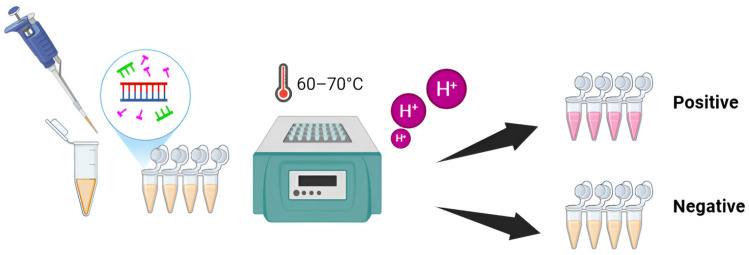
Schematic representation of NR-LAMP assay.

The colorimetric loop-mediated isothermal amplification (LAMP) assay incorporating neutral red dye was evaluated across a range of MgCl_2_ concentrations (0–10 mM) and dNTP levels (0.6–2.2 mM). Amplification temperatures ranging from 61 to 70.5 °C were refined over a 40 min period, succeeded by enzyme inactivation at 80 °C for 5 min, utilizing the CFX96 Real-Time Thermal Cycler (Bio-Rad, Hercules, CA, USA). Texas Red was used as a reporter dye for quantitative NR-LAMP assays, enabling both optimization and sensitivity assessments. Fluorescence-based amplification profiles were analyzed to determine the time to positivity for LAMP products [31]. In summary, the optimized reaction was performed in a 25 µL volume comprising 1× ammonium sulfate buffer (pH 8.8), 6 mM MgCl_2_, 1.4 mM dNTPs (SBS Genetech Co., Ltd., Beijing, China), 0.2 µM each of pol-F3 and pol-B3 primers, 0.4 µM each of pol-LF and pol-LB primers, 1.6 µM each of pol-FIP and pol-BIP primers, 0.1 mM neutral red (Invitrogen, Waltham, MA, USA), 0.32 U/µL *Bst* DNA/RNA polymerase (SBS Genetech Co., Ltd., Beijing, China), 2.9 µL RNase-free water (Apsalagen, Bangkok, Thailand), and 10 ng/µL of either proviral DNA or recombinant plasmid standard. Reactions utilizing neutral red were inspected visually for color transitions, with amplicons further validated through the observation of ladder-like banding patterns on 1.5% (*w*/*v*) agarose gel electrophoresis. A shift to pink signified a positive outcome, whereas yellow denoted a negative result (Figure 5).

### 4.6. PCR

To assess the comparative sensitivity between the LAMP assay and PCR, we analyzed extracted clinical proviral DNA samples. The PCR procedure employed the identical primer set and amplification conditions described earlier. Each reaction mixture was assembled in a final volume of 25 µL, comprising 1× DreamTaq Green PCR Master Mix (Thermo Scientific, Wilmington, NC, USA), 0.6 µM of the pol-F primer, 0.6 µM of the pol-R primer, 8.5 µL of RNase-free water (Apsalagen, Bangkok, Thailand), and 1 µL of proviral DNA derived from feline blood specimens. Following amplification, the PCR amplicons were separated via electrophoresis on a 1.5% agarose gel prepared in 1X TAE buffer (pH 8.0), subsequently stained with Novel Juice (GeneDireX, Inc., Taoyuan, Taiwan), and observed using a BluPAD LED Transilluminator (BIO-HELIX, New Taipei City, Taiwan).

### 4.7. LAMP Specificity

To evaluate the specificity of the LAMP assay, nucleic acids extracted from various feline viruses—such as feline leukemia virus (FeLV), feline coronavirus (FCoV), feline immunodeficiency virus (FIV), feline herpesvirus (FHV), feline calicivirus (FCV), and feline panleukopenia virus (FPLV)—underwent cross-amplification with the developed LAMP primers under optimized reaction conditions. A standard recombinant plasmid functioned as the positive control, whereas RNase-free water served as the negative control.

### 4.8. LAMP Detection Limit

A recombinant plasmid containing the FeLV *pol* gene was isolated as a reference standard, and its concentration was quantified using a NanoDrop spectrophotometer (Thermo Scientific, Wilmington, NC, USA). Serial tenfold dilutions were prepared in RNase-free water, ranging from 10^7^ copies/µL to 10^−1^ copies/µL. Each dilution level was analyzed in triplicate. The detection limit of the FeLV LAMP assay was established as the minimum concentration detectable through real-time fluorescence monitoring, direct visual inspection, and agarose gel electrophoresis. The number of copies per microliter was determined according to the equation provided below:

$$\mathrm{copy}\;\mathrm{number}\;\mathrm{per}\;\mu L\;=\;\frac{\mathrm{concentration}(g/\mu L)\times 6.022\;\times {\;10}^{23}}{\mathrm{nucleotide}\;\mathrm{length}\;(\mathrm{bp})\;\times \;660}$$

### 4.9. Clinical Application of LAMP Method and Statistical Analysis

To implement the colorimetric LAMP assay in a clinical setting, we collected a random selection of 98 blood samples from cats (n = 98) that had tested positive for feline leukemia virus (FeLV) via a commercial rapid diagnostic kit. These samples were obtained from the Blood Bank Unit at Kasetsart University Veterinary Teaching Hospital during a three-month interval in 2022. To assess the diagnostic performance for FeLV detection, we compared the outcomes from our developed LAMP method against those from standard clinical PCR, which served as the reference standard in this investigation. This comparison involved blinded testing, followed by the construction of a 2 × 2 contingency table. Key performance metrics, including sensitivity, specificity, positive predictive value (PPV), and negative predictive value (NPV), were calculated as percentages using IBM SPSS Statistics software (version 29.0). Additionally, Cohen’s kappa coefficient (κ) was computed and interpreted relative to the PCR results. The interpretation guidelines were as follows: κ < 0.40 indicated poor agreement, 0.41–0.60 denoted moderate agreement, 0.61–0.80 represented substantial agreement, and 0.81–1.00 signified almost perfect agreement.

## 5. Conclusions

This study successfully developed and validated a novel colorimetric neutral red-based loop-mediated isothermal amplification (NR-LAMP) assay for the detection of feline leukemia virus (FeLV). The NR-LAMP method demonstrates superior sensitivity and specificity compared to conventional PCR, achieving a detection limit of 1 copies/µL and perfect agreement with reference PCR methods for clinical samples. The assay’s innovative design, utilizing a custom primer set targeting the FeLV *pol* gene, ensures high specificity without cross-reactivity to other feline viruses. Optimizing the reaction conditions further enhanced assay efficiency and reliability. The NR-LAMP assay’s colorimetric nature allows for rapid, visual detection without the need for sophisticated equipment, making it highly suitable for resource-limited settings. The method’s cost-effectiveness, with an estimated cost of USD 1.04 per reaction, significantly reduces the financial burden compared to traditional PCR methods. This innovation not only provides a robust alternative for FeLV detection but also paves the way for broader applications in veterinary diagnostics, including longitudinal monitoring and epidemiological studies. Future work may focus on further simplifying the assay for point-of-care use and exploring its potential for detecting other viral pathogens, thereby enhancing global veterinary health surveillance efforts.

## Figures and Tables

**Table 1:** Comparative analysis of NR-LAMP and conventional PCR for FeLV detection.

NR-LAMP	Conventional PCR			Sensitivity (%) (95% CI)	Specificity (%) (95% CI)	PPV (%) (95% CI)	NPV (%) (95% CI)	κ Value
	Positive	Negative	Total					
Positive	71	0	71	97.3 (90.45–99.67)	100.0 (86.3–100)	100.0 (94.94–100)	92.6 (76.11–98.0)	0.9
Negative	2	25	27					
Total	73	25	98					

**Table 2:** Comparison of LAMP and other molecular techniques for FeLV detection.

Method	Target	LOD (copy/µL)	Amplification Time (min)	Sensitivity (%)/Specificity (%)	Ref.
NR-LAMP	*pol* gene	10^0^	40	97.3/100	This work
nPCR	U3LTR-*gag*	10^0^	>60	NA	[34]
RT-qPCR	U3LTR	8.3	44.5	NA	[35]
qPCR	U3LTR	10^3^	>60	NA	[36]
qPCR	U3LTR	180	>60	NA	[37]
RT-qPCR	LTR	NA	>60	92/99	[38]
RPA	U3LTR	NA	10 min	95.89/100	[39]

Abbreviation: LOD, limit of detection; NR-LAMP, neutral red-based loop-mediated isothermal amplification; nPCR, nested PCR; RT-qPCR, reverse-transcriptase quantitative PCR; qPCR, quantitative PCR; RPA, recombinase polymerase amplification; NA, not applicable.

**Table 3:** Service cost rate per reaction of routine PCR method in veterinary hospital in Thailand for FeLV detection.

Hospital	Cost per Reaction (USD) ^a^
Private Veterinary Hospital	29.66
Kasetsart University Veterinary Teaching Hospital	17.79
NR-LAMP (this study)	1.04

^a^ Cost was estimated based on THB prices at the time of study and was then calculated in USD.

**Table 4:** Primer detail used in this work for FeLV detection through PCR and LAMP assay. Degenerated bases were used according to IUPAC nomenclature: R (A/G), W (A/T), Y (C/T), M (A/C), K (G/T), D (A/G/T), and I (A/G/C/T).

Primer Name	Primer Sequence (5′ ⟶ 3′)	Assay	Ref.
pol-F	CYAMCCRTTATTRGGDAGAGA	PCR	[60]
pol-R	CCAGCAAGAGGTCATCTACA		
pol-F3	GCTACYGCYACTCCAATYTCC	LAMP	This work
pol-B3	GGCACMGTRGGATGGATGT		
pol-LF	GRGGYTTAATTCCYTGGTAIGC		
pol-LB	CCYGTYAARAAGCCWGGRACC		
pol-FIP (F1c-F2)	TGAGGATRCCTTGRTCYAGCA-CAGTAYCCYATGCCCCATRAR		
pol-BIP (B1c-B2)	GTCCCCATGGAATACWCCCYTA-TCTCTTAARTCYTGYACTGGT		

## Data Availability

Data are contained within the article and Supplementary Materials.

## Supplementary Materials

The following supporting information can be downloaded at https://www.mdpi.com/article/10.3390/ijms262411793/s1.
