# Supplementary material for: Innovative Colorimetric Neutral Red-Based Loop-Mediated Isothermal Amplification (NR-LAMP) Assay: Transforming Rapid and Affordable Feline Leukemia Virus Detection
Source: Int J Mol Sci. 2025 Dec 5;26(24):11793. doi: 10.3390/ijms262411793 (PMC12733238; doi:10.3390/ijms262411793)
Supplement: Supplementary file 1 [file ijms-26-11793-s001.zip › ijms-3989287-supplementary.pdf]

Supplementary Materials

Innovative Colorimetric Neutral Red-Based Loop-Mediated Isothermal Amplification (NR-LAMP) Assay: Transforming Rapid and Affordable Feline Leukemia Virus Detection

Witsanu Rapichai [1,2,3], Piyamat Khamsingnok [2], Anyalak Wachirachaikarn [4], Thawee Laodim [5], Hieu Van Dong [6], Nianrawan Meecharoen [7], Siriluk Ratanabunyong [3], Thanawat Khaoiam [2,8], Supansa Tuanthap [9], Amonpun Rattanasrisomporn [10], Selapoom Pairor [2], Kiattawee Choowongkomon [3], Natthasit Tansakul [11], Peter A. Lieberzeit [12] and Jatuporn Rattanasrisomporn [1,2,*]

1. Center for Advanced Studies for Agriculture and Food, Kasetsart University Institute for Advanced Studies, Kasetsart University, Bangkok 10900, Thailand; tswitsanu@gmail.com
2. Department of Companion Animal Clinical Sciences, Faculty of Veterinary Medicine, Kasetsart University, Bangkok 10900, Thailand; piyamat.kha@ku.th; thanawat.khao@ku.th; selapoom_ake@hotmail.com
3. Department of Biochemistry, Faculty of Science, Kasetsart University, Bangkok 10900, Thailand; ae.med@hotmail.com; fsciktc@ku.ac.th
4. Department of Sciences and Bioinnovation, Faculty of Liberal Arts and Science, Kasetsart University, Kamphaeng Saen Campus, Nakhon Pathom 73140, Thailand; faasalw@ku.ac.th
5. Department of Animal Science, Faculty of Agriculture at Kamphaeng Saen, Kasetsart University, Kamphaeng Saen Campus, Nakhon Pathom 73140, Thailand; fagrtwl@ku.ac.th
6. Faculty of Veterinary Medicine, Vietnam National University of Agriculture, Trau Quy Town, Gia Lam District, Hanoi 131000, Vietnam; dvhieuvet@vnua.edu.vn
7. Central Laboratory (CTL), Center for Veterinary Research and Innovation, Faculty of Veterinary Medicine, Kasetsart University, Bangkok 10900, Thailand; nianrawan.mee@ku.ac.th
8. Kasetsart University Veterinary Teaching Hospital, Faculty of Veterinary Medicine, Kasetsart University, Kamphaeng Saen Campus, Nakhon Pathom 73140, Thailand

[9] Faculty of Veterinary Medicine, Rajamangala University of Technology Tawan-ok, Bangpra, Chonburi 20110, Thailand; supansa_tu@rmutto.ac.th

[10] Interdisciplinary of Genetic Engineering and Bioinformatics, Graduate School, Kasetsart University, Bangkok 10900, Thailand; fgraapr@ku.ac.th

[11] Department of Pharmacology, Faculty of Veterinary Medicine, Kasetsart University, Bangkok 10900, Thailand; natthasit.t@ku.th

[12] Department of Physical Chemistry, Faculty of Chemistry, University of Vienna, Waehringer Strasse 42, A-1090 Vienna, Austria; peter.lieberzeit@univie.ac.at

* Correspondence: fvetjpn@ku.ac.th

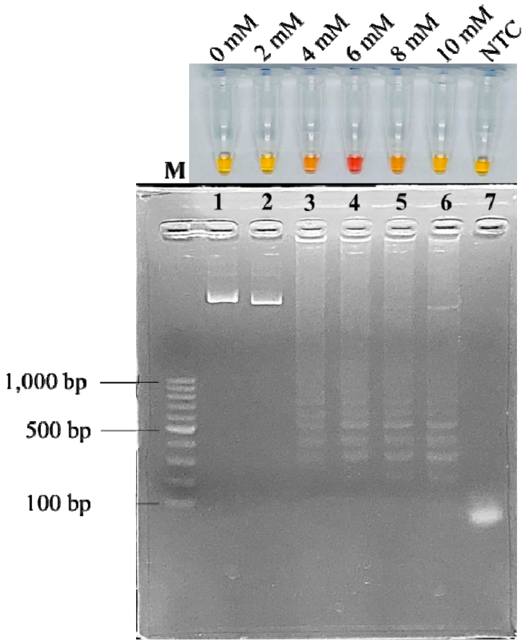

Figure S1. Effect of $MgCl_2$ concentrations on NR-LAMP assay. Pink color indicates positive reaction and yellow color indicates negative reaction. NTC, negative control. Lane M, VC 100 bp DNA Ladder (Vivantis Technologies Sdn. Bhd., Selangor Darul Ehsan, Malaysia); lane 1-6, DNA band pattern amplified at various $MgCl_2$ concentrations of 0, 2, 4, 6, 8, and 10 mM; lane 7, NTC.

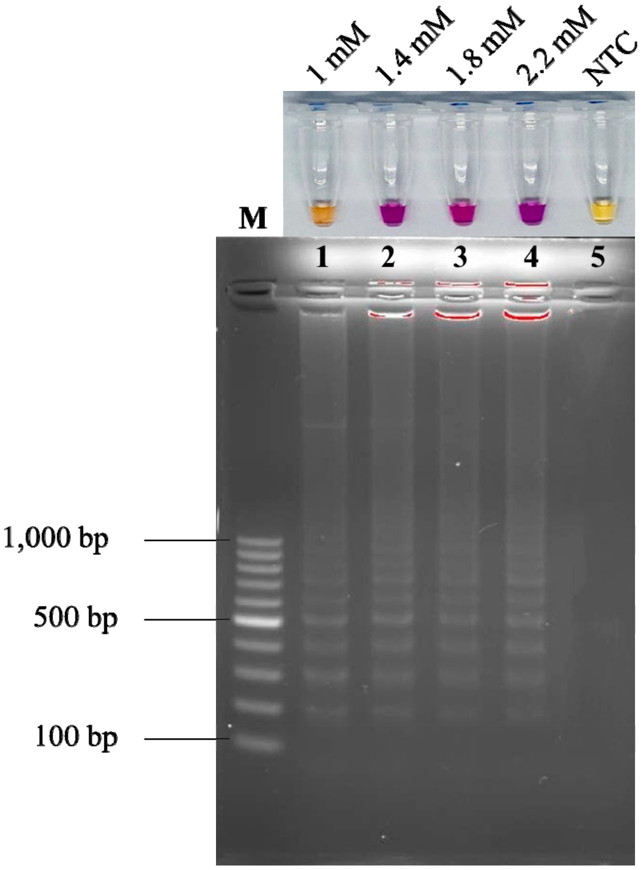

Figure S2. Effect of dNTPs concentrations on NR-LAMP assay. Pink color indicates positive reaction and yellow color indicates negative reaction. NTC, negative control. Lane M, VC 100 bp DNA Ladder (Vivantis Technologies Sdn. Bhd., Selangor Darul Ehsan, Malaysia); lane 1-4, DNA band pattern amplified at various dNTPs concentrations of 1, 1.4, 1.8, and 2.2 mM; lane 5, NTC.

Feline leukemia virus isolate C85 pol protein gene, partial cds

Sequence ID: KR030147.1 Length: **537** Number of Matches: **1**

Range 1: 1 to 537 GenBank Graphics ▼ Next Match ▲ Previo

Score	Expect	Identities	Gaps	Strand
946 bits(512)	0.0	532/541(98%)	4/541(0%)	Plus/Plus

```
Query  88   TTGCAACTAGAAGAAGAGTATCGGCTATTTGAGCCGGAAAGTGAACTAAAACAAGGTATG  147
            ||||||||||||||||||||||||||||||||||||||||||||||||||||||||||||
Sbjct  1    TTGCAACTAGAAGAAGAGTATCGGCTATTTGAGCCGGAAAGTGAACTAAAACAAGGTATG  60

Query  148  GACAGTTGGCTTAAAAACTTTCCCCAGGCATGGGCAGAAACAGGAGGTATGGGAATGGCT  207
            |||||||||||||||||||||||||||||||||||||||||||||||||| |||||||||
Sbjct  61   GACAGTTGGCTTAAAAACTTTCCCCAGGCATGGGCAGAAACAGGAGGTATAGGAATGGCT  120

Query  208  CATTGCCAAGCCCCCATCCTCATTCAACTTAAAGCTACTGCCACCCCAATCTCCATCCGG  267
            ||||||||||||||||||||||||||||||||||||||||||||||||||||||||||||
Sbjct  121  CATTGCCAAGCCCCCATCCTCATTCAACTTAAAGCTACTGCCACCCCAATCTCCATCCGG  180

Query  268  CAGTACCCCATGCCCCATGAAGCTTACCAAGGAATTAAACCCCATATAAGGAGAATGCTG  327
            ||||||||||||||||||||||||||||||||||||||||||||||||||||||||||||
Sbjct  181  CAGTACCCCATGCCCCATGAAGCTTACCAAGGAATTAAACCCCATATAAGGAGAATGCTG  240

Query  328  GACCAAGGCATCCTCAAGCCCTGCCGGTCCCCATGGAATACACCCCTATTACCTGTCAAA  387
            ||||||||||||||||||||||||| ||||||||||||||||||||||||||||||||||
Sbjct  241  GACCAAGGCATCCTCAAGCCCTGCCAGTCCCCATGGAATACACCCCTATTACCTGTCAAA  300

Query  388  AAGCCAGGAACCGGGGATTACAGACCAGTGCAGGACTTAAGAGAAGTAAATAAAAGGGTA  447
            |||||||||||||||||||||||||||||||||||||||||||||||||| |||||||||
Sbjct  301  AAGCCAGGAACCGGGGATTACAGACCAGTGCAGGACTTAAGAGAAGTAAACAAAAGGGTA  360

Query  448  GAAGACATCCATCCCACTGTGCCAAACCCATACAACCTCCTTAGCACCCTCCCACCTTCT  507
            ||||||||||||||||||||||||||||||||||||||||||||||||||||||||||||
Sbjct  361  GAAGACATCCATCCCACTGTGCCAAACCCATACAACCTCCTTAGCACCCTCCCACCTTCT  420

Query  508  CACCCTTGGTACACCGTCCTAGATCTTAAAGATGCtttttttCTGCTTGCGACTACACCC  567
            |||||||||||||||||||||||||| |||||||| ||||||||||||||||||||||||
Sbjct  421  CACCCTTGGTACACCGTCCTAGATCTAAAAGATGC-TTTTTTCTGCTTGCGACTACACCC  479

Query  568  TGAGAGCCAATTACTCTTTTGCATTTGAATGGAAAGATCCAGAAAATAGGGACTGTCAGG  627
            |||||||||||||||| |||||||||||||||||||||||||| | ||||| ||||||||
Sbjct  480  TGAGAGCCAATTACTC-TTTGCATTTGAATGGAAAGATCCAGAGA-TAGGG-CTGTCAGG  536

Query  628  G  628
            |
Sbjct  537  G  537
```

Figure S3. BLAST analysis result of clinical FeLV KU 26 sample.

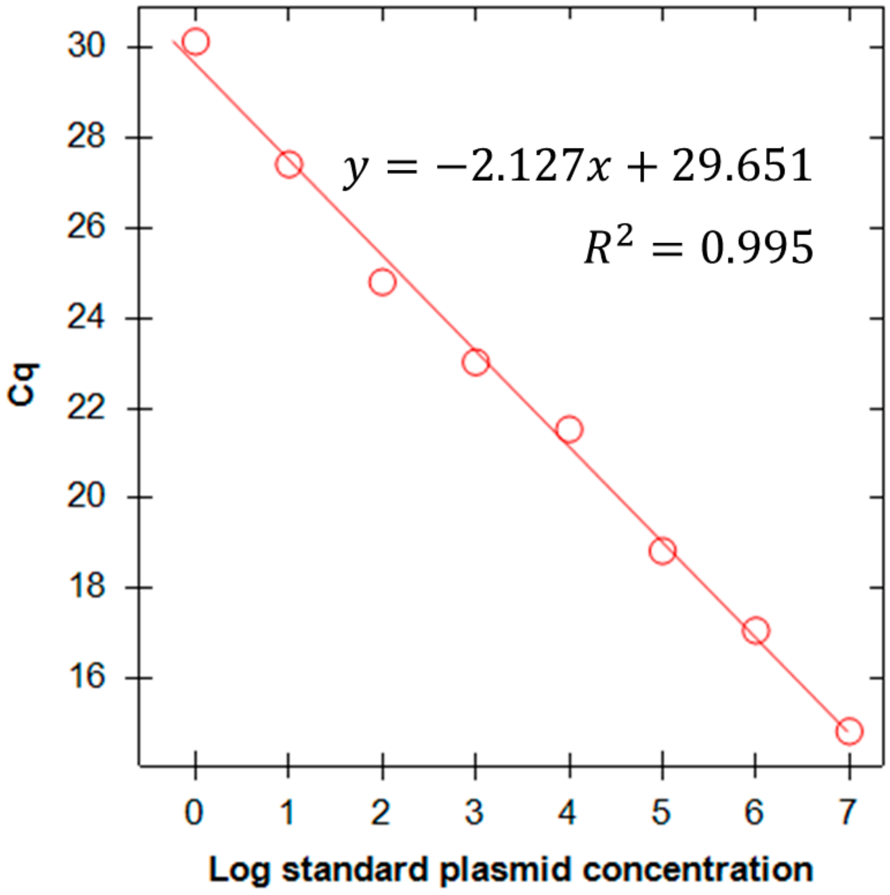

Figure S4. Calibration curve of quantitative NR-LAMP assay.

Table S1. Estimation cost per reaction of developed NR-LAMP for FeLV detection (Cost was estimated based on Thai Baht (฿) prices during performing the study, and was then calculated to US ($)).

**Component**	**Cost ($)**	**Cost per reaction ($)**
Ammonium sulfate buffer	142.73	0.0032
Magnesium chloride (Sigma)	45.87	0.021
dNTPs (Biotechrabbit)	76.74	0.027
LAMP primer set (Macrogen)	93.92	0.067
Neutral red (Invitrogen)	357.59	0.036
Bst 2.0 Warmstart DNA polymerase (New England BioLabs)	165.44	0.89
RNase free water (Apsalagen)	38.37	0.00029
Net cost		1.04
